# Research on Fault Diagnosis of Gearbox with Improved Variational Mode Decomposition

**DOI:** 10.3390/s18103510

**Published:** 2018-10-18

**Authors:** Zhijian Wang, Junyuan Wang, Wenhua Du

**Affiliations:** College of Mechanical Engineering, North University of China, Taiyuan 030051, China; wangzhijian1013@163.com (Z.W.); yihuina01161013@163.com (W.D.)

**Keywords:** gearbox, multiple fault features, permutation entropy optimization, Variational Mode Decomposition

## Abstract

Variational Mode Decomposition (VMD) can decompose signals into multiple intrinsic mode functions (IMFs). In recent years, VMD has been widely used in fault diagnosis. However, it requires a preset number of decomposition layers *K* and is sensitive to background noise. Therefore, in order to determine *K* adaptively, Permutation Entroy Optimization (PEO) is proposed in this paper. This algorithm can adaptively determine the optimal number of decomposition layers *K* according to the characteristics of the signal to be decomposed. At the same time, in order to solve the sensitivity of VMD to noise, this paper proposes a Modified VMD (MVMD) based on the idea of Noise Aided Data Analysis (NADA). The algorithm first adds the positive and negative white noise to the original signal, and then uses the VMD to decompose it. After repeated cycles, the noise in the original signal will be offset to each other. Then each layer of IMF is integrated with each layer, and the signal is reconstructed according to the results of the integrated mean. MVMD is used for the final decomposition of the reconstructed signal. The algorithm is used to deal with the simulation signals and measured signals of gearbox with multiple fault characteristics. Compared with the decomposition results of EEMD and VMD, it shows that the algorithm can not only improve the signal to noise ratio (SNR) of the signal effectively, but can also extract the multiple fault features of the gear box in the strong noise environment. The effectiveness of this method is verified.

## 1. Introduction

Gearbox is widely used in many mechanical equipment, and is the key component of equipment operation [1]. However, due to the complicated working environment and improper maintenance of the gearbox, the gearbox is prone to malfunction during its working process [2]. Due to the complexity of the internal structure of the gearbox, when a fault occurs, the fault type is mostly complex, and its fault features are often drowned in the strong background noise. Therefore, an effective feature extraction method is needed [3,4,5].

In 2014, Dragomiretskiy proposed a new signal processing algorithm, namely Variational Mode Decomposition [6]. Compared with EMD and EEMD, the algorithm has a solid theoretical foundation and high resolution accuracy [7]. Yet, the algorithm needs to set the number of decomposition layers *K* in advance, and the *K* values are often determined only by personal experience [8]. Therefore, the decomposition results are easily affected by human factors and the phenomenon of over-decomposition or under-decomposition occurs easily. That is, when *K* is too high, it will cause over-decomposition and decompose abnormal white noise components. Nevertheless, when the *K* value is too small, the phenomenon of under-decomposition will occur and some of the fault features cannot be extracted [9,10]. In addition, VMD is sensitive to noise [11], that is, the decomposition results are easily affected by the background noise, especially in the strong background noise environment, it is easier to produce false components caused by noise [12]. While for subsequent fault identification [13], the generation of false components can easily lead to misdiagnosis [14].

For the adaptive determination of the decomposition layer number *K*, Yi, and Lv are using the Particle Swarm Optimization (PSO) [15] to determine the decomposition level of *K* in VMD. Zhang and others optimize the parameters of the algorithm by using the Grasshopper Optimization Algorithm (GOA) [16]. In addition, other scholars use the ant colony algorithm (ACO) [17], artificial fish swarm algorithm (AFSA) [18], and other optimization algorithms to optimize the parameters in the VMD. Compared to the *K* value determined by personal experience, these optimization algorithms can automatically determine the *K* value based on the original signal, and have good adaptability, and the effect of human factors on the decomposition results is excluded. However, all of these algorithms are Meta-heuristic algorithms. The principle of these algorithms is a parameter optimization algorithm designed by simulating the foraging behavior of the cluster animals. In order to ensure the precision of optimization, a large population density is usually required [19].

Based on the randomness detection of the permutation entropy, this paper proposes a Permutation Entroy optimization (PEO) in order to adaptively determine the parameter *K* in VMD. The principle of the algorithm is to calculate the entropy of each intrinsic modal function decomposed by the original signal. As the anomalous component is random, its permutation entropy is much larger than the normal component [20]. Thus, after setting the threshold of the permutation entropy, whether the permutation entropy of each layer of IMF is greater than the threshold value is judged, so as to determine whether there is an abnormal component in the decomposition result, that is, whether there is an over decomposition at this time. VMD is used to decompose the original signal, until the decomposition results have abnormal components, it shows that the decomposition occurs right now, and then the *K* value at this time is reduced by one as the final optimization value.

In order to improve the signal to noise ratio(SNR) and reduce the sensitivity of VMD to noise, the author draws inspiration from the idea of Noise Aided Data Analysis (NADA) [21], and proposes a method of reducing noise based on VMD, that is, the Modified VMD (MVMD). At the same time, in order to reduce the reconstruction error and make the white noise to be completely neutralized, this algorithm uses the idea of adding white noise pairs in the Complete Ensemble Empirical Mode Decomposition (CEEMD) [22]. In each cycle, two white noises with equal amplitude and opposite sign are added to the original signal. Then the VMD is used to decompose it, and the noise in the original signal will offset each other after repeated cycles. The IMF of each layer of each cycle is integrated, and then the signal is reconstructed according to the result of the integrated mean. The reconstructed signal is decomposed by MVMD. The analysis results of simulation and experimental signals show that the decomposition result of the algorithm is better than that of VMD.

## 2. Principles of the Algorithm

### 2.1. The Principle of Permutation Entropy Algorithm

Permutation Entropy (PE) [23] is a method proposed by Bandt et al. to detect the randomness and dynamic mutation of time series. This algorithm has the advantages of simple principle, high computational efficiency, and good robustness. It is very suitable for nonlinear data analysis [24]. The specific steps of the algorithm are as follows.

Step 1: Given a discrete time series {x(i),i=1~N}. Phase space reconstructionfor each element in the time series. Get the refactoring matrix as shown in the following formula.

(1) $$\left[\begin{array}{cccc} x(1) & x(1+\tau ) & \ldots & x(1+(m-1)\tau )\\ \ldots & \ldots & \ldots & \ldots \\ x(j) & x(j+\tau ) & \ldots & x(j+(m-1)\tau )\\ \ldots & \ldots & \ldots & \ldots \\ x(K) & x(K+\tau ) & \ldots & x(K+(m-1)\tau ) \end{array}\right]$$

Among them, j=1~K,K is the number of reconstructed components. m is the embedding dimension. τ is the delay time. x(j) represents the j row component of the reconstruction matrix.

Step 2: According to the ascending rule, the reconstruction matrix of each row is arranged, and the result is shown in Equation (2).

(2) $$x(i+(j_{1}-1)\tau )\leq x(i+(j_{2}-1)\tau )\leq \ldots \leq x(i+(j_{m}-1)\tau )$$

Step 3: If there is an equal value in the component, that is, when $x(i-(j_{1}-1)\tau )=x(i-(j_{2}-1)\tau )$ occurs, then sort according to the size of the j value. That is, when $j_{1}<j_{2}$, there is

(3) $$x(i-(j_{1}-1)\tau )\leq x(i-(j_{2}-1)\tau )$$

Step 4: For every row of the reconstructed matrix, a row of symbol sequences $S(l)=(j_{1},j_{2},\ldots ,j_{m})$ can be obtained. Among them, l=1~k,k≤m!.

That is to say, in the m dimensional phase space mapping, different symbol sequences of m! group can be obtained, and S(l) belongs to one of them.

Step 5: The probability of each S(l) appears with $P_{1},P_{2},\ldots ,P_{k}$, respectively. The permutation entropy formula of the symbol sequence of k time series x(i) at different time is shown in Equation (4).

(4) $$H_{P}(m)=-\sum_{j=1}^{k}P_{j}\ln P_{j}$$

That is,

(5) $$PE_{P}(m)=H_{P}(m)=-\sum_{j=1}^{k}P_{j}\ln P_{j}$$

### 2.2. Principle of VMD

The specific construction steps of the constrained variational model are as follows.

Step 1: For the input signal x(t), through the Hilbert Transform (HT), we can get the analytic signal of each modal function $u_{k}(t)$.

Step 2: The center frequency $\omega_{k}$ of each modal function $u_{k}(t)$ is estimated, and its spectrum is moved to the baseband.

Step 3: After that, the bandwidth is estimated through the $H^{1}$ Gauss smoothness. The final constraint variational model can be expressed by Formula (6).

(6) $$\left\{ \begin{array}{l} \underset{\left(u_{k})(\omega_{k}\right)}{\min}\left\{\sum_{k}\|{\partial_{t}\left[\left(\sigma (t)+\frac{j}{\pi t})u_{k}(t\right)\right]e^{-j\omega_{k}t}\|}_{2}^{2}\right\}\\ s.t.\sum_{k}u_{k}=x(t) \end{array}\right.$$

In the equation, $\partial_{t}$ means partial derivative to *t*, $\delta_{t}$ is the impulse function, and $\left\{u_{k}\right\}=\left\{u_{1},\ldots ,u_{K}\right\}$ represents the *K* IMFs obtained by the VMD for the original signal x(t), and $\left\{\omega_{k}\right\}=\left\{\omega_{1},\ldots ,\omega_{K}\right\}$ represents the central frequency of each IMF component.

In order to solve the optimal solution of the above variational model, the following forms of Lagrange function are introduced

(7) $$\begin{array}{l} L(\left\{u_{k}\right\},\left\{\omega_{k}\right\},\lambda )=\alpha \sum_{k}{\|\left[(\sigma (t)+\frac{j}{\pi t}\right)\times u_{k}(t)]e^{-j\omega_{k}t}\|}_{2}^{2}\\ +{\|x(t)-\sum_{k}u_{k}(t)\|}_{2}^{2}+\langle \lambda (t),x(t)-\sum_{k}u_{k}(t)\rangle \end{array}$$

In the equation, λ is a Lagrange multiplier and α is a penalty factor.

Secondly, the Lagrange function of Equation (7) is transformed in time-frequency domain, and the corresponding extremum solution is carried out. The frequency domain expression of the modal function $u_{k}$ and the central frequency $\omega_{k}$ can be obtained.

(8) $${\overset{\wedge }{u}}_{k}^{n+1}(\omega )=\frac{\overset{\wedge }{f}\left(\omega \right)-\sum_{i\neq k}{\overset{\wedge }{u}}_{i}(\omega )+\frac{\overset{\wedge }{\lambda }(\omega )}{2}}{1+2\alpha {\left(\omega -\omega_{k}\right)}^{2}}$$

(9) $$\omega_{k}^{n+1}=\frac{{\int_{0}^{\infty }\omega \left|{\overset{\wedge }{u}}_{k}(\omega )\right|}^{2}d\omega }{{\int_{0}^{\infty }\left|{\overset{\wedge }{u}}_{k}(\omega )\right|}^{2}d\omega }$$

Finally, the optimal solution of the constrained variational model is solved by using the Alternate Direction Method of Multipliers (ADMM), and the original signal x(t) is decomposed into *K* IMFs.

The specific steps of the algorithm are as follows.

Step 1: The initialization of the parameters, set $\left\{{\overset{\wedge }{u}}_{k}^{1}\right\}$, $\left\{{\overset{\wedge }{\omega }}_{k}^{1}\right\}$, ${\overset{\wedge }{\lambda }}^{1}$ and n to 0.

Step 2: Update ${\overset{\wedge }{u}}_{k}$ and ${\overset{\wedge }{\omega }}_{k}$ according to Equations (8) and (9).

Step 3: Update the value of ${\overset{\wedge }{\lambda }}^{n+1}$ according to equation ${\overset{\wedge }{\lambda }}^{n+1}(\omega )={\overset{\wedge }{\lambda }}^{n}(\omega )+\tau (\overset{\wedge }{f}(\omega )-\sum_{k}{\overset{\wedge }{u}}_{k}^{n+1}(\omega ))$.

Step 4: Until the equation $\frac{\sum_{k}{\left\|{\overset{\wedge }{u}}_{k}^{n+1}-{\overset{\wedge }{u}}_{k}^{n}\right\|}_{2}^{2}}{\left\|{\overset{\wedge }{u}}_{k}^{n}\right\|}<\varepsilon$ is satisfied, the iteration is stopped and the loop is exited. Otherwise, the return step 2. Finally, *K* intrinsic mode functions can be obtained.

## 3. Improvement of VMD

Aiming at the problem of VMD, this paper adopts the permutation entropy optimization algorithm to adaptively determine the number of decomposition layers, and uses the Modified VMD to reduce the noise of the original signal and finish the final decomposition. The following two algorithms are introduced, respectively.

### 3.1. Permutation Entroy Optimization Algorithm (PEO)

First, the permutation entropy values of the following simulation signals are calculated, respectively. Among them, $x_{1}=\sin(2\times \pi \times 30)$, $x_{2}=\sin(2\times \pi \times 120)$, $x_{3}=\sin(2\times \pi \times 250)$ are sinusoidal signals. $x_{4}=(1+\cos(2\times \pi \times 12))\sin(2\times \pi \times 120)$ is an amplitude modulation signal. $x_{5}(t)=A_{m}\times \exp(-\frac{g}{T_{m}})\sin(2\pi f_{c}t)$ is a periodic shock signal, among them, $A_{m}$ = 2, damping coefficient g = 0.1, oscillation period $T_{m}$ = 0.1, natural frequency $f_{c}$ = 160 Hz. $x_{6}$ is Gauss white noise with a length of 2048.

**Figure 1:**
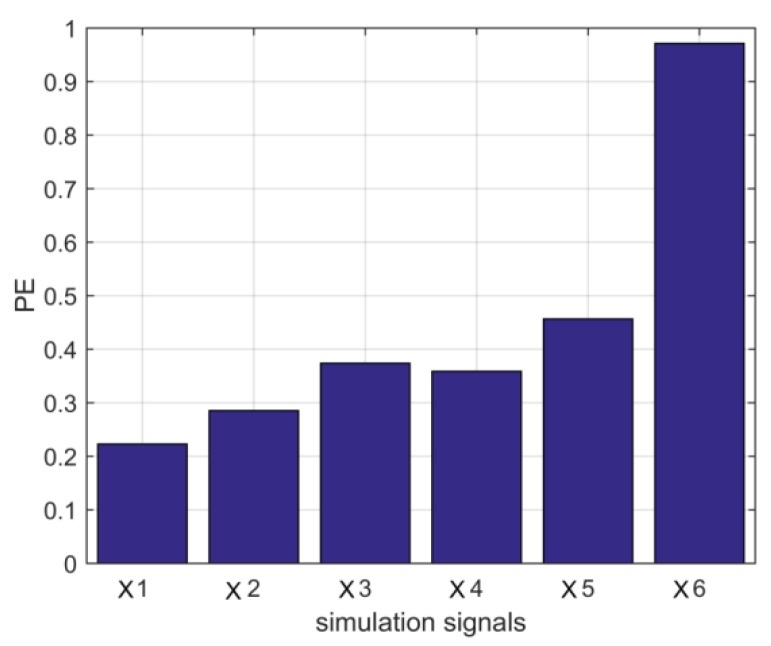
The permutation entropy value of each simulation signal.

The permutation entropy values of the above simulation signals are calculated, respectively, and the histogram as shown in Figure 1 is shown.

In order to make the experiment have better reliability, it can be known that the PE of different energy is solved. As the noise amplitude increases, PE also increases gradually, but both are greater than 0.6. When the amplitude and frequency of the modulated signal and the impulse signal change, the change of PE is small, and both are less than 0.6. As shown in Table 1, Table 2 and Table 3.

According to the above histogram, the permutation entropy of sinusoidal signal and amplitude modulation signal is small. The permutation entropy of periodic impact signals is slightly larger than that of sinusoidal and amplitude modulated signals, but neither of them exceeds the empirical threshold of 0.6. However, the permutation entropy of white noise is very large and far higher than the threshold. It shows that the Gauss white noise sequence is more random and the probability of dynamic mutation is larger, which is also consistent with the reality. Therefore, through the above analysis, we can see that according to the permutation entropy value, we can distinguish normal signals from abnormal signals. Based on the randomness detection of the permutation entropy, this paper proposes a permutation. Entroy optimization (PEO) in order to adaptively determine the parameter *K* in the VMD. The principle of the algorithm is to calculate the entropy of each intrinsic modal function decomposed by the original signal. As the anomalous component is random, its permutation entropy is much larger than the normal component. Thus, after setting the threshold of the permutation entropy, whether the permutation entropy of each layer of IMF is greater than the threshold value is judged, so as to determine whether there is an abnormal component in the decomposition result, that is, whether there is an over decomposition at this time. If not, we need to continue to increase the number of decomposition layers, that is, the number of decomposition layers needs to increase by one, and then according to the updated *K* value, the VMD is used to decompose the original signal, until the decomposition results have abnormal components, it shows that the decomposition occurs right now, and then the *K* value at this time is reduced by one as the final optimization value. The concrete steps of the algorithm are as follows:

(1) The initial value of setting *K* is 2, and the threshold of permutation entropy is taken as an empirical value 0.6.
(2) VMD is used to decompose the original signal and get *K* intrinsic mode functions ${\mathrm{imf}}_{i}(t)$ (i=1~K).
(3) The permutation entropy $pe_{i}$ (i=1~K) of each IMF in the decomposition results is calculated, respectively.
(4) Whether there is a greater than a threshold of 0.6. If there is an explanation that the decomposition result is over decomposed to cause abnormal components, then it is necessary to stop the loop and enter the step (5). If not, there is no over decomposition. The original signal also needs to increase the number of decomposition layers, that is, *K* = *K* + 1, and will return to step (2). According to the updated *K* value, we continue to decompose the original signal by VMD.
(5) The condition of the loop termination is that the current *K* value makes the decomposition result exactly the abnormal component of the permutation entropy greater than the threshold value, that is, the *K* value set at this time causes the VMD over decomposition. Therefore, it is necessary to update the *K* value before output as the final result, so that *K* = *K* − 1 can be used as the optimal solution output of the decomposition level.

### 3.2. Modified VMD (MVMD)

In order to improve signal-to-noise ratio (SNR) and reduce the sensitivity of VMD to noise, the author, inspired by the idea of Noise-Aided Data Analysis (NADA), proposed a method of noise reduction based on VMD, that is, the modified VMD (MVMD). The principle of this algorithm is to add auxiliary Gauss white noise to the original signal and make use of the uniform distribution of white noise to change the extreme distribution of the signal. After repeated cycles and integrated averages, the noise in the original signal will be greatly offset, thus achieving the purpose of homogenizing the noise in the original signal. At the same time, in order to reduce the reconfiguration error and make the white noise to be completely neutralized. This algorithm adopts the idea of adding white noise pairs in the CEEMD. That is, the white noise added in each cycle is two positive and negative white noise pairs with the same amplitude and the opposite symbol. This can guarantee the noise reduction while not increasing the new noise. After adding auxiliary white noise, two signals to be decomposed will be obtained, and then the VMD is used to decompose them, respectively. After repeated cycles, the noise in the original signal will be counterbalanced. Finally, the IMF of each layer obtained by each cycle is integrated averaging, and then the signal is reconstructed according to the result of ensemble mean. The reconstructed signal is decomposed by VMD again as the final result of MVMD. The specific steps of the algorithm are as follows:

Step 1: Initialize the parameter settings. Determine the *K* value according to the PEO algorithm. At the same time, set the number of cycles N and the amplitude of white noise Nstd.

Step 2: By adding the positive and negative Gauss white noise pairs with Nstd amplitude to the original signal, two decomposed signals $x_{i1}(t)$ and $x_{i2}(t)$ can be obtained.

(10) $$\left\{ \begin{array}{l} x_{i1}(t)=x(t)+noise_{i}(t)\\ x_{i2}(t)=x(t)-noise_{i}(t);\left(i=1\sim N\right) \end{array}\right.$$

Step 3: The two decomposed signals $x_{i1}(t)$ and $x_{i2}(t)$ are decomposed by VMD, respectively, and two groups of IMFs can be obtained. As shown by Equation (11).

(11) $$\left\{ \begin{array}{l} {\mathrm{imf}1}_{ij}(t)\;(j=1\sim K)\\ {\mathrm{imf}2}_{ij}(t)\;(j=1\sim K) \end{array}\right.$$

The ${\mathrm{imf}1}_{ij}(t)$ represents the *j*th IMF component of the signal $x_{i1}(t)$ after the *i*th decomposition, ${\mathrm{imf}2}_{ij}(t)$ represents the *j*th IMF component of the signal $x_{i2}(t)$ after the *i*th decomposition.

Step 4: Repeat steps 2 and step 3 *N* times, and add a new Gauss white noise pair at the beginning of each cycle.

Step 5: After N cycles, the final 2×N×K IMF is integrated and the result is shown in formula 12.

(12) $${\mathrm{imf}}_{j}(t)=\frac{1}{2N}\sum_{i=1}^{N}\left({\mathrm{imf}1}_{ij}(t)+{\mathrm{imf}2}_{ij}(t)\right),(j=1\sim K)$$

where ${\mathrm{imf}}_{j}(t)$ represents the ensemble mean of the *j* level IMF component in all decomposition results.

Step 6: The reconstructed signal is reconstructed according to the result of ensemble mean, as shown in Equation (13).

(13) $$x_{0}(t)=\sum_{j=1}^{K}{\mathrm{imf}}_{j}(t),(j=1\sim K)$$

Step 7: The VMD is used to decompose the reconstructed signal $x_{0}(t)$, and *K* IMFs is obtained as the final result of MVMD.

**Figure 2:**
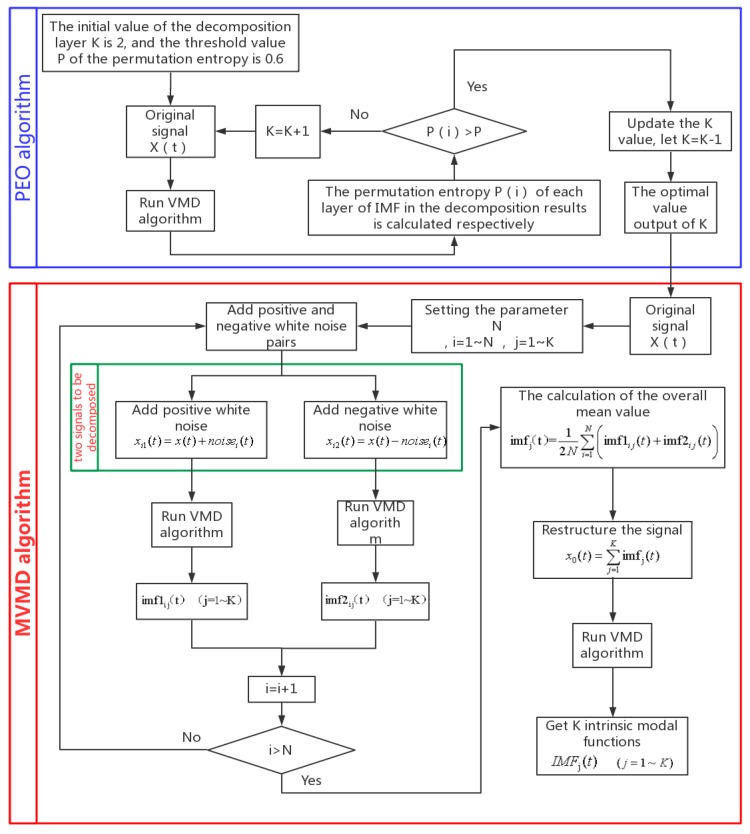
Flow chart of the Permutation Entroy Optimization- Modified Variational Mode Decomposition (PEO-MVMD).

Flow chart of MVMD based on PEO in Figure 2. The specific steps are as follows

(1) Input signal;
(2) Initialize *K* and determine the best *K* value by Permutation Entroy;
(3) Add the opposite white noise to the signal and perform MVMD decomposition;
(4) Refactoring the decomposed signal;
(5) Determine the location of the composite fault by spectrum analysis.

## 4. Simulation Signal Analysis

### 4.1. Construction of Simulation Signal

Gears and bearings are two important components, and they are also prone to fatigue damage. When the gearbox has compound faults, the vibration signals are usually with multiple modulation sources. Therefore, in the construction of the simulation signal, the simulation and analysis of the gear fault simulation signal and the rolling bearing fault simulation signal are used in this paper. The simulation signal is constructed as follows.

(14) $$x(t)=x_{1}(t)+x_{2}(t)+x_{3}(t)+0.5\times randn(t)$$

The composition signal $x_{1}(t)=2\sin(2\pi f_{1}t)$ is a sine signal. The composition signal $x_{2}(t)=(1+\cos(2\pi f_{n1}t)+\cos(2\pi f_{n2}t))\sin(2\pi f_{z}t)$ is a gear fault simulation signal containing two modulation sources, where $f_{n1}$ and $f_{n2}$ are modulation frequencies, and $f_{z}$ is the carrier frequency, that is, the meshing frequency of gears. The component signal $x_{3}(t)=A_{m}\times \exp(-\frac{g}{T_{m}})\sin(2\pi f_{c}t)$ is a periodic shock signal, which is used to simulate the fault signal of the rolling bearing, in which $A_{m}$ represents the amplitude of the shock, the g is the damping coefficient, the $T_{m}$ is the cycle of shock, and the $f_{c}$ is the rotation frequency of the bearing. The parameters are shown in the following Table 4.

**Figure 3:**
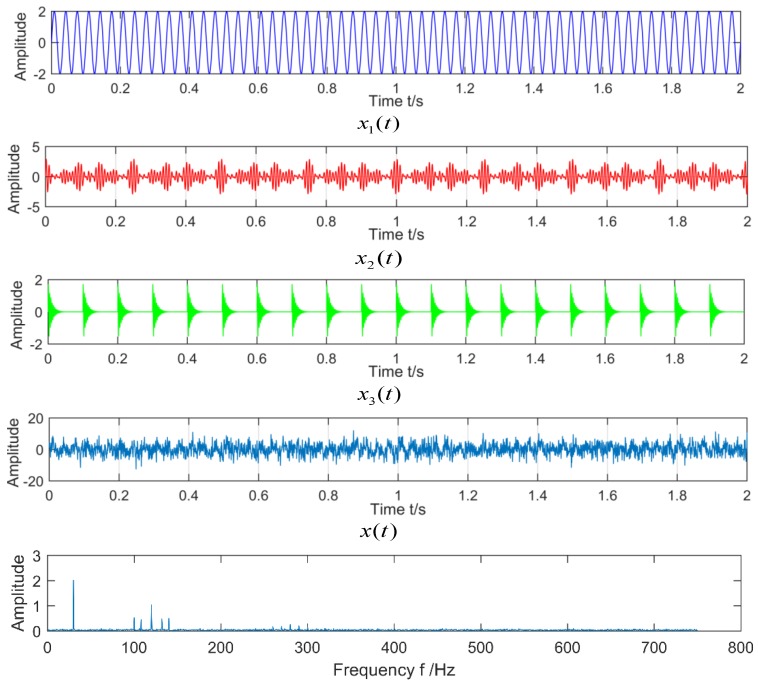
The time domain waveform of each component signal and its spectrum.

Set the number of sampling points *N* to 3000, and the sampling frequency is 1500 Hz. The time-domain waveforms of the component signal $x_{1}(t)$, $x_{2}(t)$, $x_{3}(t)$, and the simulation signal x(t) are shown in Figure 3. Through the spectrum analysis, the carrier frequency and the natural frequency of the impact signal in the composite fault signal can be extracted. However, the natural frequency has a small amplitude and is easily disturbed by noise.

### 4.2. Adaptive Determination of Decomposition Layer K by PEO Algorithm

**Figure 4:**
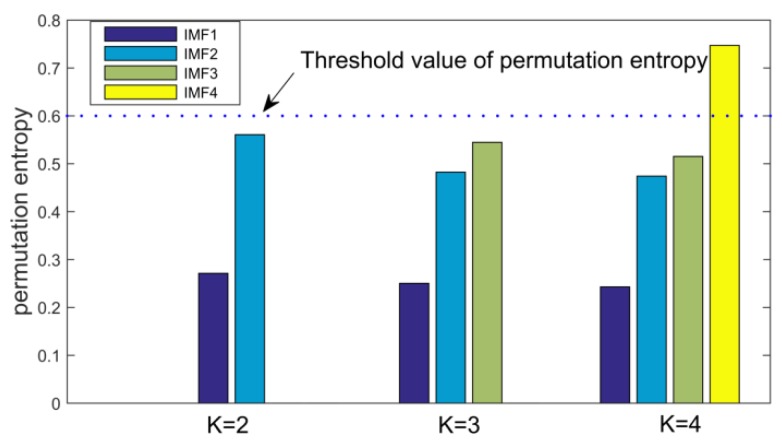
Permutation entropy of Intrinsic Mode Functions (IMF) in each iteration of PEO algorithm with different *K* value in the VMD.

First, we use the PEO algorithm to determine the decomposition level *K*, set the initial value of *K* to two. Find out the optimal value of *K* according to whether or not there is over decomposition. After each cycle iteration, the permutation entropy of each IMF of the original signal decomposed by VMD is calculated. As shown in the following Figure 4.

According to the running result of the PEO algorithm, it is known that when *K* = 4, the decomposition results appear the abnormal component of permutation entropy value greater than the threshold value, while the *K* = 3 does not appear abnormal components, which indicates that the *K* = 4 happens to be over decomposition, so the decomposition mode number *K* of VMD is three. The number of components in the simulation signal is also exactly three, which is consistent with the operation results of the PEO algorithm, which shows that the PEO algorithm can indeed adaptively determine the optimal value of the *K*.

### 4.3. Parameter setting of MVDM

**Figure 5:**
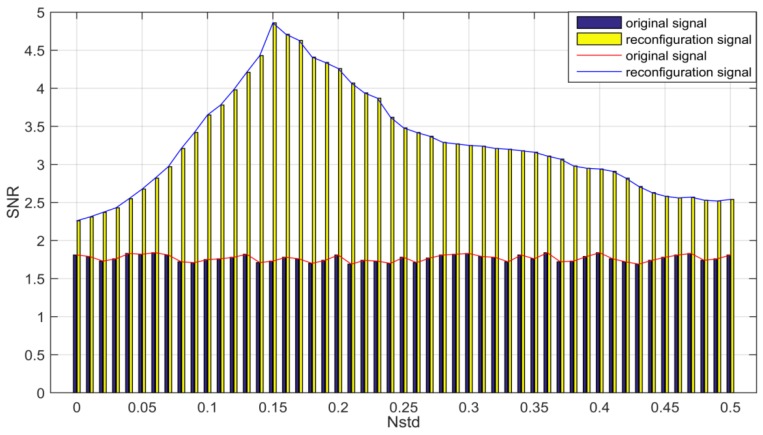
SNR-Nstd (Signal to noise ratio -Noise of the standard deviation of the added) diagram.

In the MVMD, we need to set the number of cycles *N* and the white noise amplitude *N*std added. The greater the number of cycles, the better the effect of homogenization noise. However, the efficiency of signal processing needs to be taken into account. This paper takes the number of cycles *N* = 100. For the selection of the amplitude of positive and negative Gauss white noise, the signal to noise ratio (SNR) of reconstructed signal is chosen as the basis for choosing the amplitude of white noise. Through a lot of experiments, the experimental results are drawn into the SNR-Nstd diagram as shown in Figure 5.

It is known from the diagram that when the white noise amplitude *N*std is 0.15, the signal to noise ratio of the reconstructed signal is the highest, that is to say, the effect of noise reduction is the best. Therefore, for the simulation signal, the amplitude of white noise added in the MVMD is 0.15.

### 4.4. Comparison of Decomposition Results of Different Algorithms

**Figure 6:**
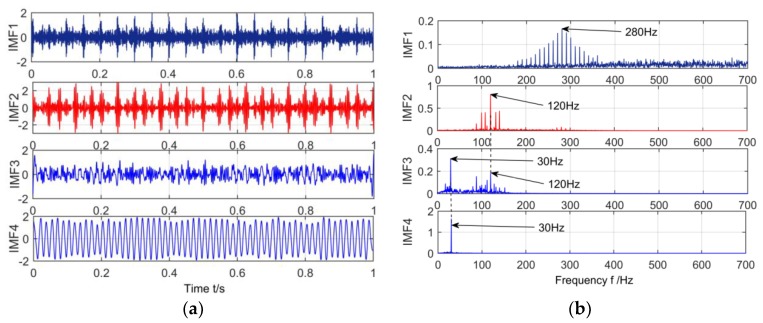
The spectrum of IMFs after Ensemble Empirical Mode Decomposition (EEMD) and its corresponding spectrum. (**a**) Time domain of IMFs after EEMD; (**b**) The spectrum corresponding to each layer of IMF.

In order to achieve transversal contrast between different EEMD, VMD, and MVMD are used to decompose the simulation signals, respectively. In the decomposition results of EEMD, only the first four layers with strong correlation with the original signal are analyzed. The corresponding decomposition results of each algorithm are shown in Figure 6.

It can be seen from Figure 6 that there is a serious modal aliasing phenomenon in EEMD decomposition, and 120 Hz and 30 Hz are, respectively, decomposed into two different feature components.

**Figure 7:**
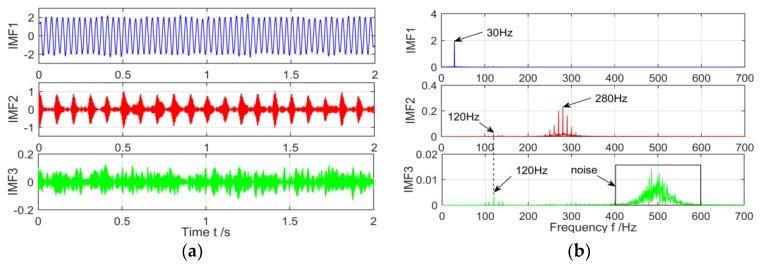
The spectrum of IMFs after VMD decomposition and its corresponding spectrum. (**a**) Time domain of IMFs after VMD; and (**b**) The spectrum corresponding to each layer of IMF.

The decomposition results of the VMD is shown in Figure 7, the frequency spectrum of the decomposition can be found that the low frequency components of the 30 Hz are successfully extracted from the original signal, and the frequency spectrum is less affected by the noise. However, due to the interference of strong background noise, the modal aliasing occurs in the 120 Hz signal, which is decomposed into the two modes of IMF2 and IMF3. The characteristics of the spectrum are very weak.

**Figure 8:**
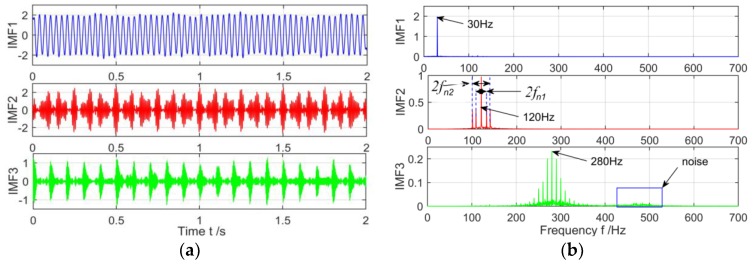
The spectrum of IMFs after MVMD decomposition and its corresponding spectrum. (**a**) Time domain of IMFs after MVMD; and (**b**) The spectrum corresponding to each layer of IMF.

Finally, the decomposition results of the MVMD is shown in Figure 8, it is found that the spectrum characteristics of the low frequency signal of the 30 Hz in the original signal are very obvious by observing the time spectrum of the IMF1. In IMF2, the central frequency 120 Hz of the amplitude modulation signal and the two modulation frequencies $f_{n1}$ and $f_{n2}$ are also successfully stripped from the original signal with strong noise, and the side band is evenly distributed on both sides of the main frequency. In IMF3, The center frequency 280 Hz and the uniform distribution on both sides of the 10 Hz side are also very obvious. While the noise components appear near 500 Hz, the noise components are very weak compared to the main frequency components of the 280 Hz, which has little influence on the identification of the fault features. In addition, compared to Figure 6 and Figure 7, the MVMD can not only effectively eliminate the phenomenon of modal aliasing in VMD, but also obtain very obvious frequency characteristics in the strong noise environment.

## 5. Analysis of Measured Signal in Gear Box

In order to verify the effectiveness and feasibility of the MVMD in engineering practice, the relevant experiments on closed power flow gearbox test rig are carried out in this paper. The complex fault vibration signals of the gear box under the condition of normal, tooth surface pitting and bearing outer ring are measured respectively. Then, the MVMD is used to process these complex fault vibration signals, and a good extraction effect is obtained. The effectiveness and feasibility of the proposed method are verified.

**Figure 9:**
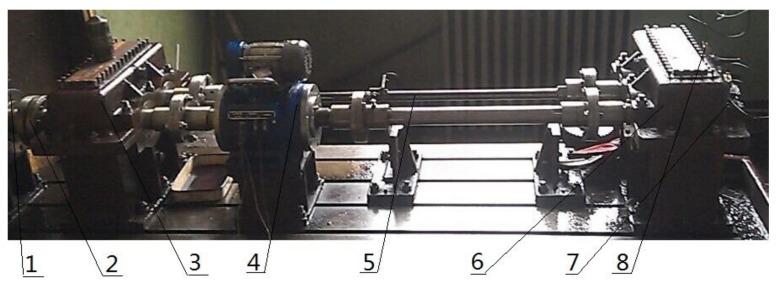
Gearbox test rig. 1—Speed regulating motor; 2—Clutch; 3—Companion gearbox; 4—Rotating speed torsion meter; 5—Torsion bar; 6—Test gear box; 7—Triaxial acceleration sensor 1#; 8—Triaxial acceleration sensor 2#.

In this experiment, the closed power flow test rig is used to collect the compound fault signal of gearbox. In the experiment, the gear box was loaded by the internal force generated by the torsion bar. The speed of gearbox is adjusted by controlling the electromagnetic speed regulating asynchronous motor, and the regulating range is 120 r/min–1200 r/min. The test rig is shown in Figure 9, where the fault bearing is at the three direction acceleration sensor 1#.

The experimental device mainly includes the speed display, the three direction acceleration sensor YD77SA (sensitivity is 0.01 V/ms^2^), the test gear, the test bearing 32,212, the motor, the rotating shaft and so on. The specific experimental parameters are shown Table 5.

**Figure 10:**
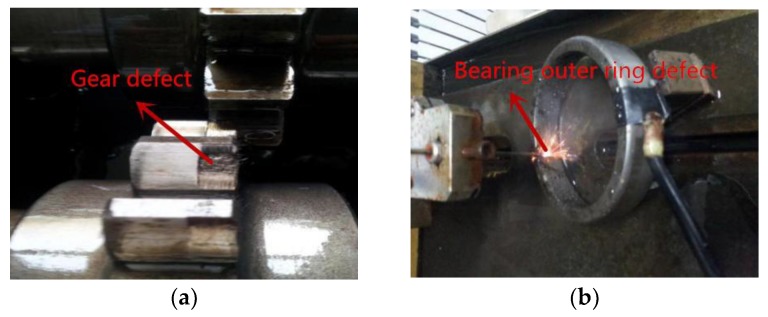
Figure of gear and bearing outer ring fault. (**a**) Gear pitting failure; and (**b**) Implantation of outer ring fault by EDM.

In order to verify the feasibility and effectiveness of the above methods, the fault type of gear box in this experiment is set up as multiple faults. The composite fault types include pitting corrosion and outer ring fault of the bearing, as shown in Figure 10.

When the gear system produces vibration shock, the vibration signal will be transmitted to the shaft first, then the shaft will be transferred to the bearing, and finally transmitted to the gearbox. When the acceleration sensor is placed, the location of the sensor should be as close to the vibration source as it can reduce the attenuation of the fault characteristics in the transmission process, so the best position of the measuring point should be the bearing seat. Therefore, in this experiment, two acceleration sensors 1# and 2# are arranged in this experiment, and the two sensors are used to measure the vibration signals of three directions of X, Y, and Z on two bearing seats, respectively. As shown in Figure 9, the acceleration sensor 1# is arranged on the bearing seat of the failure bearing, and the acceleration sensor 2# is arranged on the bearing seat of the normal bearing. The vibration signal used in this experiment comes from the acceleration sensor 1#.

**Figure 11:**
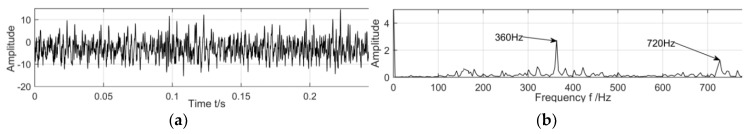
Time-frequency spectrum of complex fault signal of gear box. (**a**) Time domain of complex fault vibration signal; and (**b**) Spectrum of complex fault vibration signal.

The vibration signal of compound fault of gearbox collected by closed power flow test rig is shown Figure 11. The units of amplitude is mm/s^2^.

The time-frequency spectrum of complex fault signal of gearbox shows that due to the influence of strong background noise, the waveform of time domain is chaotic and irregular. In the spectrum, the gear meshing frequency 360 Hz and its two doubling 720 Hz are appeared, and the fault frequency of the outer ring does not appear, so the fault signal needs further decomposition. VMD and the MVMD based on PEO will be used to decompose the above complex fault signals respectively.

First, the PEO is used to determine the decomposition level *K*, and the initial value of *K* is set to 2. The iteration is iterated and the optimal value of *K* is found. The optimal value of the *K* output by the final PEO algorithm is two, so the number of decomposition modes is *K* = 2. In addition, through a large number of experiments, the amplitude of the white noise added in the MVMD is 0.85, and the number of cycles is *N* = 100.

**Figure 12:**
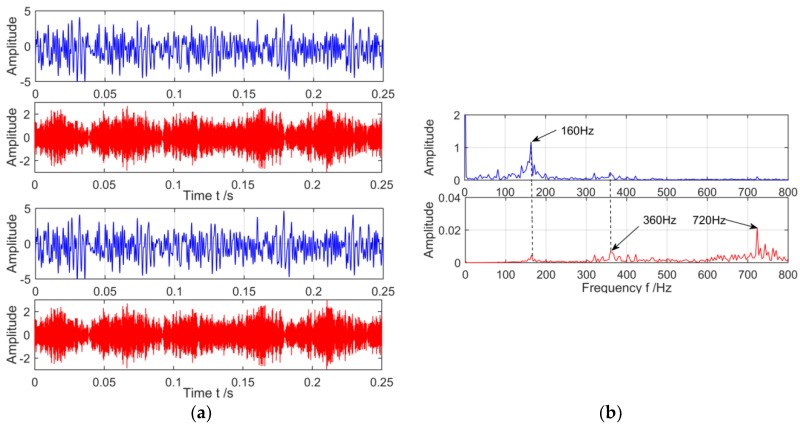
The spectrum of IMFs after VMD decomposition and its corresponding spectrum. (**a**) Time domain of IMFs after VMD of vibration signal; and (**b**) The spectrum corresponding to each layer of IMF.

**Figure 13:**
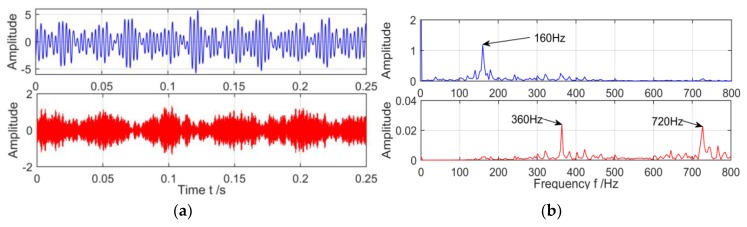
The spectrum of IMFs after MVMD decomposition and its corresponding spectrum. (**a**) Time domain of IMFs after MVMD of vibration signal; and (**b**) The spectrum corresponding to each layer of IMF.

Secondly, in order to form a lateral contrast, the VMD and MVMD will be used to decompose the above gearbox compound fault signals. The two algorithm decomposes the fault signal as shown in Figure 12 and Figure 13.

As shown in Figure 12 as the result of the decomposition of VMD, it is found by observing the spectrum that for the complex fault features of the gear box under the strong background noise, the VMD has a poor extraction effect on the feature, and the two frequency components in the signal all have serious modal aliasing, which makes this very weak fault feature more difficult to be extracted. This has caused great difficulties in judging the types of faults. For the decomposition results of the MVMD, the fault frequency of the outer ring in the gearbox 160 Hz, the gear fault characteristic frequency 360 Hz and its two frequency doubling 720 Hz are successfully extracted, and the effect is very obvious compared with the VMD.

## 6. Conclusions

VMD requires a preset number of decomposition layers *K* and is sensitive to background noise. These shortcomings also become a bottleneck in the practical application of the algorithm. Therefore, an improved algorithm based on Variational Mode Decomposition is proposed in this paper. Through the analysis of the simulation signal and the experimental signal of the gear box, the experimental results show that compared with the VMD, the proposed PEO based MVMD has more obvious advantages. It not only overcomes the limitations of the VMD, but also successfully extracts the complex fault features of the gear box under the strong background noise. The validity and feasibility of this method are verified. In the future, the parameters can be intelligently determined to optimize the VMD and improve the decomposition accuracy. Consider optimizing information entropy and fuzzy entropy as the objective function to further optimize VMD.

## Figures and Tables

**Table 1:** The permutation entropy values corresponding to different noise amplitude.

Amplitude	0.2	0.5	0.8	1	1.5	2
PE	0.6524	0.7832	0.8231	0.8937	0.9435	0.9846

**Table 2:** The permutation entropy values corresponding to different frequency of modulation signal.

Frequency	80	120	180	240	300	360
PE	0.3822	0.3835	0.3876	0.3926	0.3935	0.3921

**Table 3:** The permutation entropy values corresponding to different amplitude of impulse signal.

Amplitude	1.5	2	2.5	3	3.5	4
PE	0.4824	0.4821	0.4838	0.4872	0.4905	0.4931

**Table 4:** The parameters of the simulation signal.

$f_{1}$	$f_{n1}$	$f_{n2}$	$f_{z}$	$A_{m}$	g	$T_{m}$	$f_{c}$
28 Hz	12 Hz	20 Hz	120 Hz	2	0.1	0.1	280

**Table 5:** Experimental parameters.

transmission ratio	1:1
engagement system	Half-tooth meshing
frequency of samplingFs	8000 Hz
Sampling point N	2000
load troque T	1000 N·m
Gear tooth number z	18
rotational speed n	1200 rpm
rotor frequency $f_{n}$	20 Hz
Bearing outer ring fault frequency	160.2 Hz
Gear meshing frequency	180 Hz
